# Effect of different levels of hydrolysable tannin intake on the reproductive hormones and serum biochemical indices in healthy female rats

**DOI:** 10.1038/s41598-020-77672-0

**Published:** 2020-11-26

**Authors:** Faiza Manzoor, Mahr Un Nisa, Hafiz Amjad Hussain, Nazir Ahmad, Huma Umbreen

**Affiliations:** 1 Faculty of Life Sciences, Institute of Home and Food Sciences, Government College University, Faisalabad, 38000 Punjab Pakistan; 2 Department of Internal Medicine, Faisalabad Medical University, Faisalabad, 38000 Punjab Pakistan

**Keywords:** Molecular biology, Cardiology, Diseases, Endocrinology, Gastroenterology

## Abstract

The present work aimed to find out the effect of different levels of hydrolysable tannin (HT) on serum hormonal profile, biochemical indices, lipid profile, apparent digestibility of nutrients and body weight gain in healthy female rats. Forty five adult healthy female rats of 8 weeks old were randomly divided into five equal groups. Different doses of HT 0, 0.5, 1, 1.5 and 2% were administered daily to each rats group on the body weight basis for 6 weeks. The results had shown the reduction trend (*p < *0.05) in the blood glucose, serum cholesterol, low density lipoprotein, testosterone, prolactin, ghrelin, total oxidative stress and serum iron levels; while an improvement (*p < *0.05) was seen in serum follicle stimulating hormone, progesterone, luteinizing hormone, high density lipoprotein, IgM and total antioxidant capacity. However, no effect (*p > *0.05) was noticed in serum IgG, protein, estrogen and calcium levels. A significant reduction (*p < *0.05) was seen in the apparent nutrient digestibility and body weight gain. The results had shown improvement in the feed conversion ratio (*p < *0.05) but non-significant decrease (*p > *0.05) in the feed intake. The findings showed that HT had healthy effects on the serum biochemical indices and reproductive hormonal profile but had a negative impact on the nutrient digestibility. Thus, the study concluded that HT could be used as an herbal medicine for the treatment of leading metabolic and infertility diseases like obesity and polycystic ovarian syndrome in females.

## Introduction

The metabolic disorders like obesity, hypertension, diabetes, dyslipidemia and cardiovascular diseases are the biggest health concerns today, associated with the high intake of western diets. It is assumed that the development of polycystic ovarian syndrome and infertility disorders in females are also due to the poor eating habits and obesity^1^. The preventive measures are being taken to fight against these metabolic health disorders by using different medicines but researchers have tried their best to find better substitutes of these drugs with fewer side effects which are of great interest. The studies have shown that polyphenols are proved to be very effective to address such diseases. Among these phyto-nutrients, tannins have shown many health promoting properties like antiviral, anti-inflammatory, immune modulator and antioxidant^2^. On the basis of the structural differences, tannins are divided into two groups as hydrolysable tannins (HT) and condensed tannins (CT). The later are oligomers and polymers composed of flavon-3-ol nuclei, having higher molecular weight of 1000–20 Da. Derivatives of flavan-3-ols like catechin, epicatechin and gallocatechin are the main basic units of CT while the HT contains a central core of glucose or polyol esterified with gallic acid having molecular weight of 500–3000 Da^3^. The HT, on the basis of hydroxyl group, is further classified into gallic acid (gallotannins) and ellagic acid (ellagitannins) which possess different biological activities^4^. It also plays an important role against acute toxicity developed by oxidative stress (OS)^5^. The OS is the state of production of free radicals which is one of the major causes for the development of metabolic disorders; thus, HT decreases the state of OS due to its strong antioxidant potential^6^. Most of its forms also help to improve the glucose and insulin resistance and fight against diabetes in animal research models^7^. The HT has shown some anti-nutritional properties especially in mono-gastric animals’ nutrition forming complexes with protein, carbohydrates and starches. The strength of these positive and negative effects of HT on digestion was observed when used in different quantities; fewer intakes have shown no significant impact on digestibility of crude protein, organic matter and ash in animals’ trials^8^. Higher intake of HT decreases the daily feed intake, gain in weight^9^ and final body weight^10^. So, the previous studies proved that HT had shown both healthy as well as detrimental effects. However, no such study was planned which aimed to find out the healthy as well as the anti-nutritional effects of HT on the blood biochemical indices in healthy female rats which might be useful for the treatment of metabolic disorders. Secondly, this study was also aimed to rationalize the use of HT for the management of female hormonal and infertility disorders in relation to their secondary metabolite profiles so that some natural therapeutic alternatives might be discovered.

## Materials and methods

### Animals

Forty five adult female Wistar Albino rats of 8 weeks old were selected in this trial with an average body weight of 135 ± 5 g. They were kept at 25 ± 1 °C with 45 to 55% of humidity with light and dark cycle of 12-h. The rats were housed in metabolic cages for the collection of feces. The total duration of this trial was 57 days, out of which 15 days were for diet adjustment and 42 days were of HT intake period. The rats were completely randomized into five groups (n = 9), named as per HT dose levels i.e. Ht0: group with 0% HT; Ht0.5: group with 0.5% HT; Ht1: group with 1% HT; Ht1.5: group with 1.5% HT; Ht2: group with 2% HT. Each rat was given an identification mark using permanent ink markers of different colors on their tail^11^. All procedures were performed according to the guide lines for the management of the Laboratory Animal^12^, approved by the Animal Ethical Committee of Government College University, Faisalabad, Pakistan.

### Dose preparation

The HT powder was sponsored by Diversified Marketing Group (DMG, Rawalpindi). The different doses of HT 0, 0.5, 1, 1.5 and 2% per kg body weight were calculated for each rat by using the formula^13^: Dosage in mg = Body weight of the rat (g)/1000 g × dose (mg). The calculated doses of HT/rat/day/kg of body weight were dissolved in warm water of required volume^14^. The groups of female rats were offered the prepared solutions once in a day between 10 to 11 am.

### Chemical composition of diet

All experimental female rats were fed with *iso-caloric* and *iso-nitrogenous* diet as shown in Table 1. The standard diet contained all the required vitamins and minerals as per AIN-93 guidelines^12,15^. Rats were fed food and water ad libitum. The blood glucose (Accu-Check Glucometer, Bayer) and body weight (Weighing Balance) of each rat were monitored on weekly basis.

**Table 1:** The ingredients of diet (g/kg) and their nutrient contents (%).

Ingredients	Amount(g/1000 g)	Nutritive value of diet on dry matter basis (%)	
Corn starch	230	Dry matter	88.2
Maltodextrine	100	Moisture	11.8
Sucrose	100	Ash	8.7
Soya bean meal	420	Crude fat	5.9
Maize bran	50	Crude fiber	7.2
Soyabean oil	50	Crude protein	18.9
Methionine	3	Nitrogen free extract	47.5
AIN 93G Vit Mix	10		
AIN 93G Min Mix	35		
Choline Bitartarate	2		
Total Calories(kcal)	3822		

### Feed intake (g)

The feed given to each group was weighed and recorded on daily basis. Split and leftover feed were also measured in order to get the exact estimation of daily feed intake and feed conversion ratio^16^.

$$ {\text{Feed}}\;{\text{conversion}}\;{\text{ratio}}\left( {\% {\text{FCR}}} \right) = {\text{Consumption}}\;{\text{of}}\;{\text{Feed}}\left( {\text{g}} \right)/{\text{Body}}\;{\text{weight}}\;{\text{gain}}({\text{g}}) $$

Gain in Body weight (BWG) was measured by using formula^17^: Final weight (g) – Initial weight (g).

### Apparnt nutrient digestibility (%)

In the last 7 days of the trial, the daily feces were collected, weighed, pooled and stored at − 20 °C to determine the apparent digestibility of nutrients^18^.

$$ {\text{Apparent}}\;{\text{Nutrient}}\;{\text{Digestibility}}\left( \% \right) = \frac{{{\text{Nutrient}}\;{\text{Intake}}\;\left( {{\text{g}}/{\text{day}}} \right) - {\text{Nutrient}}\;{\text{in}}\;{\text{Feces}}\;\left( {{\text{g}}/{\text{day}}} \right)\; \times {1}00}}{{{\text{Nutrient}}\;{\text{Intake}}\left( {{\text{g}}/{\text{day}}} \right)}} $$

The Official Methods of Analysis^19^ were used for the estimation of dry matter, crude fat, protein, fiber and ash in feed and feces. The dry matter (DM) was determined when the samples were dried at 65 °C for 48 h by using a hot air oven. The ash contents were determined by incineration at 550 °C for 4 h in a Muffle Furnace and Petroleum Ether Extraction method was used for the estimation of crude fat (EE). The crude fiber (CF) was determined by digestion method with H_2_SO_4_ and NAOH and the crude protein contents (CP) by Kjeldahl Method while the carbohydrates (%NFE) were measured by deduction as [100 − % (moisture + crude fat + crude protein + crude fiber + ash)] ^20^.

### Experimental protocols

The female rats were kept overnight in fasting (12 h). Three replicates of each group were made and two rats from each replicate were sacrificed early morning at 8:00 o’clock using chloroform for anesthesia. The neck area exposing the jugular vein was cut with the sharp scalpel blade. Blood was collected into the test tubes and allowed to clot. The serum had been obtained after centrifugation at 5000 rpm/twenty minutes and stored at − 20 °C for the biochemical analysis.

### Serum biochemical profile

Serum samples for cholesterol (mg/dl) and triglycerides (mg/dl) were analyzed spectrometrically with Enzymatic Calorimetric Method with lipid clearing factor (LCF) after enzymatic hydrolysis and oxidation (CHOD-PAP) (Human Diagnostic Worldwide, Netherland); Serum HDL (mg/dl) was determined enzymatically (DiaSys Diagnostic System GmbH, Germany) serum LDL (mg/dl) was calculated as difference using Friedewald Eq ^21^:

$$ {\text{LDL}} - {\text{cholesterol}} = {\text{Total}}\;{\text{cholesterol}}\left( {{\text{mg}}/{\text{dl}}} \right){-}\left( {{\text{Triglyceride}}\left( {{\text{mg}}/{\text{dl}}} \right)/{5}} \right){-}{\text{HDL}} - {\text{cholesterol}}({\text{mg}}/{\text{dl}}) $$

For the detection of total antioxidant capacity (TAC; mmol of Trolox _equiv/L_) Erel Method was used^22^. The standard curve was made with standard 0.1, 0.3, 0.6, 0.9, 1.2 and 1.5 mmol/L from Trolox (Vitamin E analog; 30m*M*^stock^). Minimum detectable range 0.18 mmol/L and was up to 6 mmol of Trolox _equiv/L_ with intra-assay CV under 3%. Total antioxidant status (TOS; µmol of H_2_ O_2equiv_.∙L^−1^) was measured in serum samples by Erel Method^23^ in which the TOS was determined with the equivalent standards of H_2_O_2_ (12.50, 6.25, 3.12, and 1.56 μM/L) from the standard curve. Minimum range was calculated from standard curve 0.13 µmol of H_2_O_2equiv/L_ and linearity up to 200 µmol of H_2_O_2equiv/L_ with intra-assay CV which was kept under 10%. Serum IgG (g/l) and IgM (g/l) were assessed by Bindarid Radial Immune-Diffusion (RID) kit method in which antigen diffused radially into the agarose gel that contained appropriate mono specific antibody. Antigen–antibody complexes formed a precipitin ring. The ring size was increased until an equilibrium was reached between the formation and breakdown of these complexes. A linear relationship existed between the square of the ring diameter and the antigen concentration. By measuring the ring diameter, a calibration curve was constructed (The Binding Site Ltd., Birmingham, UK). Serum protein was analyzed spectrometrically with Biuret Method^24^. Serum iron (µg/dl) was determined by performing Photometric Colorimetric Test with iron lipid clearing factor (LCF) by using commercially available kit (Human Diagnostic worldwide, Netherland). Serum calcium (mg/dl) was determined by Arsenazo III Colorimetric Method^25^.

### Serum hormonal profile

Serum follicular stimulating hormone (FSH; mlµ/ml), prolactin (ng/ml), progesterone (ng/dl) and testosterone (ng/dl) were determined by Radioimmunoassay (RIA; Gamma Counter) using the kits of Beckman Coulter, Inc. USA. The Enzyme-Linked Immune-sorbent Essay (ELIZA) Method was used for the determination of serum luteinizing hormone (LH mlµ/ml) using the kit of Pointe Scientific Inc. USA; serum insulin (µlU/ml) was determined by using kit of Calbiotech Inc. USA; serum estrogen (pg/ml) was determined by using kit of ALPCO, USA; serum leptin (ng/ml) and ghrelin (ng/ml) were determined by using kits of Elabscience Biotechnology Inc. Corporate, USA (Eliza Reader).

### Statistical analysis

Data expressed in Mean ± SEM and statistically significant differences were calculated by Analysis of Variance (ANOVA) using *IBM SPSS Statistics 21*(USA) at *p* value < 0.05 and < 0.01.

## Results

### Feed intake, body weight gain (g), feed conversion ratio and nutrient digestibility (%)

The effects of different levels of HT on feed intake (FI), body weight gain (BWG), feed conversion ratio (FCR), apparent nutrient digestibility and true digestibility are shown in Table 2, 3 and 4 respectively. No change (*p > *0.05) was seen in average FI of all the experimental groups as compared to Ht0. The HT had shown a reduction trend (*p < *0.05) in the BWG of Ht0.5 (14.75 g), Ht1 (13.13 g), Ht1.5 (12.24 g) as compared to Ht0 (16.04 g). However, a significant reduction (*p < *0.01) was noticed in Ht2 (10.14 g) as compared to Ht0. An improvement (*p < *0.05) was noticed in FCR of Ht1.5 (1.70%) and Ht2 (2.06%) as compared to Ht0.5 (1.43%), Ht1 (1.60%) and Ht0 (1.33%) as shown in Table 2. After HT intervention the digestibility of dry matter, ash, crude fat, protein and fiber are presented in Table 3. A decrease in dry matter (*p < *0.05) was seen in Ht1.5 (66.27%) and Ht2 (65.22%) as compared to Ht0.5 (71.15%), Ht1 (70.25%) and Ht0 (70.50%). Crude protein was also highly significantly reduced (*p < *0.01) in Ht2 (72.30%) as compared to Ht0 (79.82%); while the Ht0.5 (76.51%), Ht1 (71.07%) and Ht1.5 (76.53%) were non-significant to each other but significantly reduced (*p < *0.05) in comparison with Ht0. The digestibility of crude fat was reduced (*p < *0.05) in Ht1.5 (67.65%) and Ht2 (68.36%) as compared to Ht1, Ht0.5 and Ht0 as shown in Table 3.The effect of HT on digestibility of crude fiber had shown reduction (*p < *0.05) in Ht2 (68.64%), Ht1 (69.69%) and Ht1.5 (70.15%) as compared to Ht0.5 (72.17%) and Ht0 (71.69%). Ash contents were highly significantly decreased (*p < *0.01) in Ht2 (66.34%) compared to Ht0 (74.68%) while Ht0.5 (71.97%), Ht1 (72.90%) and Ht1.5 (70.22%) were non-significant to each other but reduced (*p < *0.05) as compared to Ht0. Nitrogen free extract was highly significantly decreased (*p < *0.05) in Ht2 (44.65%) and Ht1.5 (45.28%) as compared to Ht0.5 (49.68%), Ht1(49.09%) and Ht0 (49.40%) as shown in Table 3.

**Table 2:** Feed intake, body weight gain (g) and feed conversion ratio (%) of healthy female rats.

Groups	Ht0	Ht0.5	Ht1	Ht1.5	Ht2
Average FI (g/day)	21.24 ± 0.12^a^	21.05 ± 0.09^a^	21.02 ± 0.09^a^	20.98 ± 0.09^a^	20.95 ± 0.09^a^
Average BWG (g)	16.04 ± 0.050^a^	14.75 ± 0.027^b^	13.13 ± 0.028^c^	12.42 ± 0.041^d^	10.14 ± 0.026^e^
FCR (%)	1.33 ± 0.009^e^	1.43 ± 0.015^d^	1.60 ± 0.015^c^	1.70 ± 0.018^b^	2.06 ± 0.017^a^

*FI* Feed intake, *BWG* Body Weight Gain, *FCR* Feed Conversion Ratio.

**Table 3:** Apparent nutrient digestibility (%) in healthy female rats.

Nutrients %	Groups				
	Ht0	Ht0.5	Ht1	Ht1.5	Ht2
Dry matter	70.50 ± 0.26^b^	71.15 ± 0.78^a^	70.25 ± 0.74^ab^	66.27 ± 0.66^c^	65.22 ± 0.67^c^
Crude protein	79.82 ± 0.53^a^	76.51 ± 0.54^b^	77.07 ± 0.35^b^	76.53 ± 0.69^b^	72.30 ± 0.76^c^
Crude fat	74.22 ± 0.62^ab^	75.05 ± 0.95^a^	71.93 ± 0.78^b^	67.65 ± 0.90^d^	68.36 ± 0.54^c^
Crude fiber	71.69 ± 0.72^b^	72.17 ± 0.48^a^	69.69 ± 0.38^c^	70.15 ± 0.34^bc^	68.64 ± 0.73^d^
Ash	74.68 ± 0.61^a^	71.97 ± 0.29^ab^	72.90 ± 0.92^b^	70.22 ± 0.52^bc^	66.34 ± 0.69^c^
Nitrogen free extract	49.40 ± 0.85^a^	49.68 ± 0.78^a^	49.09 ± 0.56^a^	45.28 ± 0.54^b^	44.65 ± 0.41^c^

Means sharing similar letter in a Rows are statistically non-significant (*P > *0.05).

**Table 4:** True nutrient digestibility value of feces.

	Groups				
	Ht0	Ht0.5	Ht1	Ht1.5	Ht2
**Nutrient % in Feces**					
Dry matter	57.3	58.5	59.5	61.2	61.5
Ash	7.8	7.9	8.1	8.3	8.4
Crude fat	5.1	5.3	5.7	5.7	5.9
Crude fiber	6.8	6.8	6.9	6.9	7.1
Crude protein	13.9	14.1	14.4	14.6	14.9
Nitrogen free extract	23.7	24.4	24.4	26.1	26.2

### Blood glucose (mg/dl)

The results regarding weekly blood glucose levels after the intake of HT, in different groups of healthy female rats are shown in Table 5. A reduction trend in weekly blood glucose was observed in all the groups from 4th week as compared to Ht0. The weekly blood glucose levels were reduced (*p < *0.05) in the last experimental week in Ht2 (88.77 mg/dl) and Ht1.5 (90.33 mg/dl) as compared to Ht0.5 (92.49 mg/dl), Ht1 (92.05 mg/dl) and Ht0 (94.05 mg/dl) as shown in Table 5. The average blood glucose levels of Ht1.5 (91.92 mg/dl) and Ht2 (91.35 mg/dl) had shown significant reduction (*p < *0.05) as compared to Ht0.5 (93.45 mg/dl), Ht1 (93.04 mg/dl) and Ht0 (94.74 mg/dl).

**Table 5:** Weekly blood glucose levels (mg/dl) of healthy female rats.

Weeks	Reference value^26^ Groups					Mean
	Ht0	Ht0.5	Ht1	Ht1.5	Ht2	
W1	94.73 ± 1.39	94.31 ± 1.88	94.51 ± 2.64	94.36 ± 1.37	94.31 ± 1.87	94.44 ± 0.71^a^
W2	94.45 ± 1.33	94.28 ± 1.10	93.63 ± 2.17	92.51 ± 0.92	92.50 ± 1.64	93.47 ± 0.61^a^
W3	94.44 ± 0.54	93.70 ± 1.53	93.30 ± 1.90	92.28 ± 1.42	91.97 ± 1.70	93.14 ± 0.61^a^
W4	94.46 ± 0.26	93.39 ± 2.00	92.67 ± 1.51	91.56 ± 0.91	90.97 ± 2.18	92.53 ± 0.66^ab^
W5	94.35 ± 0.20	92.52 ± 1.59	92.07 ± 1.50	90.46 ± 1.48	89.57 ± 1.74	91.74 ± 0.68^b^
W6	94.05 ± 0.90	92.49 ± 1.45	92.05 ± 1.81	90.33 ± 1.16	88.77 ± 0.90	91.54 ± 0.69^b^
Mean	94.74 ± 0.32^a^	93.45 ± 0.58^a^	93.04 ± 0.71^ab^	91.92 ± 0.54^b^	91.35 ± 0.74^b^	

Means sharing similar letter in a row or in a column are statistically non-significant (*p > *0.05).

### Serum biochemical profile

Table 6 shows the serum biochemical profile of Ht0 and after the intake of different levels of HT in Ht0.5, Ht1, Ht1.5 and Ht2 of healthy female rats. A reduction (*p < *0.05) was observed in serum cholesterol, low density lipoprotein (LDL) and triglycerides (TG); while improvement (*p < *0.05) in serum high density lipoprotein (HDL) was observed as compared to healthy reference value. The serum cholesterol level was reduced significantly (*p < *0.05) in Ht1.5 (99.34 mg/dl) and Ht2 (95.79 mg/dl) as compared to Ht0.5 (108.01 mg/dl), Ht1 (105.18 mg/dl) and Ht0 (108.19 mg/dl). The serum LDL had shown reduction (*p < *0.05) in Ht1 (45.76 mg/dl) and Ht1.5 (40.07 mg/dl) as compared to Ht0.5 (50.32 mg/dl) and Ht0 (51.07 mg/dl); while significant decrease (*p < *0.01) was observed in Ht2 (36.44 mg/dl) as compared to Ht0.5, Ht1, Ht1.5 and Ht0. The serum HDL level of Ht1 (39.42 mg/dl), Ht1.5 (39.91 mg/dl) and Ht2 (40.35 mg/dl) were non-significant to each other but significantly increased (*p < *0.05) as compared to Ht0.5 (37.39 mg/dl) and Ht0 (36.62 mg/dl). The serum TG level was significantly decreased (*p < *0.05) in Ht2 (97.90 mg/dl) as compared to Ht0.5, Ht1, Ht1.5 and Ht0 as shown in Table 6. Serum protein and IgG had shown non-significant effects (*p > *0.05). Serum IgM level was improved significantly (*p < *0.05) in Ht1.5 (0.82 g/l) and Ht2 (0.89 g/l) as compared to Ht0.5 (0.76 g/l), Ht1 (0.78 g/l) and Ht0 (0.74 g/l). The serum calcium had shown no change (*p* > 0.05). However, serum iron level was reduced significantly (*p < *0.05) in Ht2 (227.75 µg/dl) as compared to Ht0.5 (232.27 µg/dl), Ht1 (230.90 µg/dl), Ht1.5 (229 µg/dl) and Ht0 (234.51 µg/dl). The HT had a significant effect on TAC and TOS levels in healthy female rats. The serum level of TAC had shown a significant improvement (*p < *0.05) in Ht1 (3.85 mmol/L), Ht1.5 (3.88 mmol/L) and Ht2 (3.89 mmol/L) as compared to Ht0.5 (3.80 mmol/L) and Ht0 (3.80 mmol/L). The serum TOS was reduced (*p < *0.05) in Ht1 (18.65 µmol/L), Ht1.5 (18.07 µmol/L) and Ht2 (17.86 µmol/L) as compared to Ht0.5 (19.70 µmol/L) and Ht0 (19.93 µmol/L) as shown in Table 6.

**Table 6:** Serum biochemical profile of healthy female rats.

Groups	Reference value	Ht0	Ht0.5	Ht1	Ht1.5	Ht2
Cholesterol (mg/dl)	80–114^27^	108.19 ± 0.65^a^	108.01 ± 0.40^a^	105.18 ± 0.40^b^	99.34 ± 0.28^bc^	95.79 ± 0.53^c^
HDL (mg/dl)	38–63^27^	36.62 ± 0.62^b^	37.39 ± 0.32^b^	39.42 ± 0.47^a^	39.91 ± 0.53^a^	40.35 ± 0.44^a^
LDL (mg/dl)	32–86^27^	51.07 ± 0.50^a^	50.32 ± 0.60^b^	45.76 ± 0.38^b^	40.47 ± 0.24^bc^	36.44 ± 0.67^c^
TG (mg/dl)	64–129^27^	102.16 ± 0.10^a^	101.79 ± 0.32^a^	100.76 ± 0.32^b^	100.00 ± 0.40^b^	97.90 ± 0.38^c^
Protein (g/dl)	5.6–7.6^28^	5.66 ± 0.019^a^	5.67 ± 0.013^a^	5.67 ± 0.020^a^	5.68 ± 0.015^a^	5.67 ± 0.026^a^
IgG (g/l)	3.09–3.41^29^	3.27 ± 0.012^a^	3.28 ± 0.006^a^	3.28 ± 0.006^a^	3.29 ± 0.003^a^	3.30 ± 0.006^a^
IgM (g/l)	0.59–0.91^29^	0.74 ± 0.015^c^	0.76 ± 0.006^bc^	0.78 ± 0.009^bc^	0.82 ± 0.012^b^	0.89 ± 0.029^a^
Ca (mg/dl)	5.3–13^28^	11.15 ± 0.024^a^	11.16 ± 0.020^a^	11.16 ± 0.023^a^	11.17 ± 0.019^a^	11.17 ± 0.023^a^
Fe (ug/dl)	230–275^30^	234.51 ± 0.69^a^	232.27 ± 0.92^b^	230.90 ± 0.89^bc^	229 ± 0.35^cd^	227.75 ± 0.36^d^
TAC(mmol/L)	3.81–3.99^31^	3.80 ± 0.010^b^	3.80 ± 0.006^b^	3.85 ± 0.040^a^	3.88 ± 0.041^a^	3.89 ± 0.044^a^
TOS (µmol/L)	17.52–20.72^31^	19.93 ± 0.23^a^	19.70 ± 0.57^b^	18.65 ± 0.63^c^	18.07 ± 0.26^c^	17.86 ± 0.11^d^

*HDL* high density lipoprotein, *LDL* low density lipoprotein, *TG* Triglycerides, *IgG* Immunoglobulin G, *IgM* Immunoglobulin M, *TAC* total anti oxidant capacity, *TOS* total oxidative status.

### Serum hormonal profile

The effects of different levels of HT on serum hormonal profile of healthy female rats are presented in Table 7. No change (*p* > 0.05) was noticed in serum levels of insulin and leptin hormones while a reduction trend (*p < *0.05) was seen in the levels of serum ghrelin in Ht1.5 (174.82 ng/ml) and Ht2 (174.73 ng/ml) as compared to Ht0.5, Ht1 and Ht0 (174.95 ng/ml). Serum testosterone levels of Ht1.5 (1.41 ng/dl) and Ht2 (1.38 ng/dl) were highly significantly reduced (*p < *0.05) in comparison with Ht0 (1.46 ng/dl); while Ht0.5 (1.43 ng/dl) and Ht1 (1.42 ng/dl) were shown non-significant effects (*p* > 0.05) as shown in Table 7. No change (*p* > 0.05) in serum estrogen level was observed. An improvement (*p < *0.05) was observed in the level of serum LH in Ht1.5 (1.58mlµ/ml) and Ht2 (1.64mlµ/ml) as compared to Ht0.5 (1.51mlµ/ml), Ht1 (1.52mlµ/ml) and Ht0 (1.54mlµ/ml). Serum FSH levels of Ht1.5 (2.64 mlµ/ml) and Ht2 (2.67mlµ/ml) were also improved significantly (*p < *0.05) as compared to Ht0.5 (2.57 mlµ/ml), Ht1 (2.57 mlµ/ml) and Ht0 (2.56 mlµ/ml). An increase (*p < *0.05) was noticed in serum progesterone level of Ht2 (26.33 ng/dl) as compared to Ht0 (25.61 ng/dl), while Ht0.5, Ht1 and Ht1.5 had shown non-significant effects (*p* > 0.05). However, a decline (*p < *0.05) was seen in serum prolactin levels of Ht1.5 (28.78 ng/dl) and Ht2 (27.70 ng/dl) as compared to Ht0.5, Ht1 and Ht0 (30.25 ng/dl) as shown in Table 7.

**Table 7:** Serum hormonal profile of healthy female rats.

Groups	Reference value	Ht0	Ht0.5	Ht1	Ht1.5	Ht2
Insulin (µlU/ml)	13–18^32^	15.01 ± 0.16^a^	15.02 ± 0.17^a^	15.02 ± 0.10^a^	15.05 ± 0.08^a^	15.04 ± 0.11^a^
Leptin (ng/ml)	6–8^33^	6.50 ± 0.02^a^	6.52 ± 0.02^a^	6.53 ± 0.03^a^	6.52 ± 0.02^a^	6.54 ± 0.01^a^
Ghrelin (ng/ml)	150–200^34^	174.95 ± 0.04^a^	174.94 ± 0.03^a^	174.94 ± 0.03^a^	174.82 ± 0.05^b^	174.73 ± 0.06^b^
Estrogen (pg/ml)	27–34^35^	33.77 ± 0.18^a^	33.77 ± 0.18^a^	33.76 ± 0.18^a^	33.77 ± 0.08^a^	33.77 ± 0.13^a^
Testosterone(ng/dl)	1.24–1.54^35^	1.46 ± 0.00^a^	1.43 ± 0.01^ab^	1.42 ± 0.01^b^	1.41 ± 0.00^bc^	1.38 ± 0.02^c^
LH (mlµ/ml)	1.4^35^	1.54 ± 0.01^b^	1.51 ± 0.02^b^	1.52 ± 0.05^b^	1.58 ± 0.01^ab^	1.64 ± 0.03^a^
FSH (mlµ/ml)	2.59 ^36^	2.56 ± 0.00^b^	2.57 ± 0.00^b^	2.57 ± 0.00^b^	2.64 ± 0.03^a^	2.67 ± 0.00^a^
Progesterone(ng/ml)	22.47^37^	25.61 ± 0.03^b^	25.62 ± 0.02^b^	25.63 ± 0.03^b^	25.65 ± 0.03^b^	26.33 ± 0.34^a^
Prolactin (ng/ml)	33.23^38^	30.25 ± 0.01^a^	29.86 ± 0.33^a^	29.49 ± 0.33^ab^	28.78 ± 0.33^b^	27.70 ± 0.33^c^

The subscript numbers in most left column indicate the reference value cited in respective studies. FSH: Follicle stimulating hormone; LH: Luteinizing Hormone.

## Discussion

Hydrolysable tannin (HT) is made up of carbohydrates, mainly D-glucose in their central core, which is used for the farm animals’ production and improves their health status. Previous studies also proved that HT had shown anti-diabetic, anti-obesity, anti-inflammatory, antiviral and antioxidants activity in various animal models. The present study was aimed to find out the protective and anti-nutritional effects of HT on healthy female rats. The first finding in present study was that HT had decreased significantly the digestibility of protein and weight gain. The present data was also co-related with previous results that the utilization of tannin had significantly reduced the protein digestion and weight gain as compared to low tannin diet^39^. The previous studies reported that tannin intake had reduced the protein digestion by forming the tannin-protein complexes which were not easily absorbed and eventually resulted weight loss^40^. The FCR was also increased in the present trial which might be due the anti-nutritional property of the HT which was responsible for poor feed utilization due to remarkably low weight gain^41^. Different studies reported that the consumption of various kinds of tannin had also reduced the feed intake or had shown no effect on it^42^. The HT had shown non-significant decrease in feed intake which might be due to the poor digestibility of the dry matter and decreased serum ghrelin level which helped to reduce feed intake; while on the other hand the decreased fiber digestibility was observed in the present trial which helped to improve feed intake, so the effect on intake was non-significant in the present study. However, the serum ghrelin level might be reduced due to the poor fiber digestibility as reported previously^43^. The first biochemical results after oral administration of HT had shown a significant decrease in blood glucose while no effect was observed on insulin level when compared with control, which was also reported in previous studies^44^.The possible mechanism through which HT had decreased the glucose level might be the activation of the insulin signaling pathway in adipocytes^45^ or it might have enhanced the insulin activity^46^. Another possible reason of the decreased glucose level might be the decline in the glucose absorption from intestine^46^ due to the decrease in the digestibility of carbohydrates which was also observed in the present trial. As reported previously^44,47^ in different research trials that poor digestibility of the carbohydrate might be due to the formation of complexes of tannin with starch digestive enzymes which further inhibited the process of digestion; while no change in insulin level was observed in the present trial due to the extra pancreatic effect of tannin which was independent of insulin^46^ or the HT had insulin like activity that maintained the antioxidant environment of the pancreatic β-cells^48^. Another statistical finding of present trial was the decreased levels of serum LDL, cholesterol and triglycerides and increased level of serum HDL which was also reported previously^47^. The intake of HT had also improved serum lipid profile which might possibly be due to the binding of tannin with fat excreted in the form of fecal bile and by increasing the cholesterol catabolism greater than that of its synthesis in the liver^49^ or it might be decreased due to the poor digestibility of fat which was also another finding of this study. A decrease was noticed in nutrient digestibility of crude fiber and ash content which was also reported previously^40^. The digestive tract was one of the main sites for the anti-nutritional effect of the tannin^38^ and possible mechanism of the decreased nutrient digestibility might be due to the changes involved in the permeability of the intestinal wall. As a result of this reaction between HT and the membrane protein of the intestinal mucosal cells, there appeared less nutrient absorption^42^. In the present trial the serum leptin had shown non-significant effects which might be due the fact that the experimental rats were healthy. As previously mentioned^6^ that HT was a biological antioxidant which helped to reduce the production of free radicals which were present in excess quantity into the reproductive tracts of males and females subjects and caused OS and further enhanced the infertility problems^50^. So the decreased level of OS was required to maintain the reproductive health and hormonal balance which was another finding of the present trial and a significant increase in TAC and significant decrease in TOS were also observed in experimental rats after six weeks of tannin intake and it was also reported in previous study^51^. So an improvement was observed in the reproductive hormones like LH, FSH and progesterone of female rats whereas a significant decrease was observed in serum testosterone and prolactin levels in the present trial, which might be due to the reduction in TOS that further improved the reproductive hormones. However, no effect was observed on serum estrogen which might be due to the fact that the rats were healthy and there was no disturbance present in the estrous cycle of female rats. The HT possessed antiviral and anti-microbial properties which improved the body immune system^52^. The present study had also found a significant increase in the immunoglobulin IgM which was correlated with the previous works that ellagic acid, a form of HT, had improved the serum IgG and IgM in infected mice^53^. But in another study, the intake of tannin had decreased the level of IgG and IgM in growing chicks^54^. However, no change in IgG level was observed in present study due to the healthy experimental rats. In addition, the increased IgM level might be due to the reason that HT had acted as an immune-modulator substance which enhanced the serum globulin level^55^ and this increase might contribute to further increase in serum immunoglobulin IgM level. Serum protein was the bio-marker of the nutritional status while no change was observed in serum protein levels with different levels of tannin intake as previously reported in many studies^40^. Hence, it meant that tannin might not have a toxic effect on the liver protein synthesis as well as on the metabolism of dietary protein. Another finding of present study was the decreased serum iron level which was also previously reported in many studies and the possible mechanism of this decrease was due to the binding of tannin with iron^56^.

## Conclusion

The results proved that 1.5% and 2% levels of HT had shown strong antioxidant potential which produced a healthy and beneficial impact on the serum biochemcal indices and also improved the reproductive hormonal profile like FSH, LH, progesteron, prolactin and testosterone. Therefore, HT had the theraputic potential for the management of the metabolic and hormonal disorders like diabeties, cardiovascular risk factors, obesity and might be used for the treatment of polycystic ovarian syndrome.

## References

[1] Hajivandi L (2020). Food habits in overweight and obese adolescent girls with polycystic ovary syndrome (PCOS): a qualitative study in Iran. BMC Pediatrics.

[2] Saxena M (2013). Phytochemistry of medicinal plants. J. Pharmacol. Phytol..

[3] Smeriglio A (2016). Proanthocyanidins and hydrolysable tannins: occurrence, dietary intake and pharmacological effects. Brit. J. Pharmacol..

[4] Mueller-Harvey I (2001). Analysis of hydrolysable tannins. Anim. Feed. Sci. Technol..

[5] Agarwal A, Gupta S, Sharma KR (2005). Role of oxidative stress in female reproduction. Reprod. Biol. Endocrinol..

[6] Frankič T, Salobir J (2011). In vivo antioxidant potential of Sweet chestnut (Castanea sativa Mill.) wood extract in young growing pigs exposed to n-3 PUFA-induced oxidative stress. J. Sci. Food. Agric..

[7] Hsu C, Yen G (2007). Effect of gallic acid on high fat diet-induced dyslipidaemia, hepatosteatosis and oxidative stress in rats. Br. J. Nutr..

[8] Addisu S (2016). Effect of dietary tannin source feeds on ruminal fermentation and production of cattle; a review. J. Anim. Feed. Res..

[9] Schiavone A (2008). Effects of a natural extract of chestnut wood on digestibility, performance traits, and nitrogen balance of broiler chicks. Poult. Sci..

[10] Jamroz D (2009). Effect of sweet chestnut tannin (SCT) on the performance, microbial status of intestine and histological characteristics of intestine wall in chickens. Br. Poult. Sci..

[11] Silverman J, Suckow MA, Murthy S (2014). The IACUC handbook.

[12] Nutrient Requirements of Laboratory Animals: Fourth Revised Edition (n.d.). Retrieved from http://www.nap.edu/catalog/4758/nutrient-requirements-of-laboratory-animals-fourth-revised-edition (1995) 25121259

[13] Erhirhie E, Ekene NE, Ajaghaku D (2014). Guidelines on dosage calculation and stock solution preparation in experimental animals’ studies. J. Nat. Sci. Res..

[14] Bonelli F (2018). Oral administration of chestnut tannins to reduce the duration of neonatal calf diarrhea. BMC. Vet. Res..

[15] Reeves PG, Nielsen FH, Fahey GC (1993). AIN-93 purified diets for laboratory rodents: final report of the American institute of nutrition ad hoc writing committee on the reformulation of the AIN-76A rodent diet. J. Nutr..

[16] Manjula K (2016). Feed efficiency and serobiochemical profile of wistar rats fed with spirulina as functional food. Curr. Res. Nutr. Food. Sci. J..

[17] Yao J (2019). Hydrolysable tannin supplementation alters digestibility and utilization of dietary protein, lipid, and carbohydrate in grass carp (ctenopharyngodonidellus). Front. Nutr..

[18] Shi H (2015). Effects of replacing wild rye, corn silage, or corn grain with CaO-treated corn stover and dried distillers grains with solubles in lactating cow diets on performance, digestibility, and profitability. J. Dairy. Sci..

[19] AOAC (Association of Official Analytical Chemists). Official Methods of Analysis. 15th ed. Assoc. *Off. Anal. Chem.* Arlington, VA (1990).

[20] Adenike K (2013). Effect of processing on the lectin and trypsin inhibitor content of Plukenetia conophora seeds as it affects growth performance and nutrients metabolism in rat. Afr. J. Food. Sci..

[21] Kaur J (2014). Use of friede wald equation for dyslipidemia in metabolic syndrome. Int. J. Med..

[22] Erel O (2004). A novel automated direct measurement method for total antioxidant capacity using a new generation, more stable ABTS radical cation. Clin. Biochem..

[23] Erel O (2005). A new automated colorimetric method for measuring total oxidant status. Clin. Biochem..

[24] Gornall AC, Bardawill CJ, David MM (1949). Determination of serum proteins by means of the Biuret Reaction. J. Biol. Chem..

[25] Bauer PJ (1981). Affinity and stoichiometry of calcium binding by arsenazo III. Anal. Biochem..

[26] Konsue A (2017). Fasting blood glucose levels and hematological values in normal and streptozotocin-induced diabetic rats of Mimosa pudica L. extracts. Pharm. J..

[27] Ihedioha JI (2013). Reference values for the serum lipid profile of albino rats (Rattusnorvegicus) of varied ages and sexes. Comp. Clin. Pathol..

[28] Johnson-Delaney, C. A. *Exotic Companion Medicine Handbook for Veterinarians*. Wingers Publishing Incorporated (1996).

[29] Obiazi H (2017). Effect of moringa oleifera leaf extract on immunoglobulins of albino wistar rats. Int. J. Sci. Res..

[30] Wojciak RW (2014). Alterations of selected iron management parameters and activity in food-restricted female Wistar rats (animal anorexia models). Eat. Weight. Disord..

[31] Turgat F (2008). Antioxidant and protective effects of silymarin on ischemia and reperfusion injury in the kidney tissues of rats. Int. Urol. Nephrol..

[32] Koksal B (2015). Effect of streptozotocin on plasma insulin levels of rats and mice: a meta-analysis study. Open Access Macedonian. J. Med. Sci..

[33] Zou B (2014). Persimmon tannin accounts for hypolipidemic effects of persimmon through activating of AMPK and suppressing NF-κB activation and inflammatory responses in High-Fat Diet Rats. Food Funct..

[34] Johnson ML (2016). Plasma Ghrelin Concentrations Were Altered with Oestrous Cycle Stage and Increasing Age in Reproductively Competent Wistar Females. PLoS ONE.

[35] Badawi AM (2018). The possible protective effect of Bougainvillea spectabilis leaves extract on estradiol valerate-induced polycystic ovary syndrome in rats (biochemical and histological study). Eur. J. Anat..

[36] Yakubu MT (2014). Effects of aqueous extract of cissuspopulnea stem on function indices of the ovary and uterus of female wistar rats. Centr. J..

[37] Zia, M. S. *et al*. Effect of monosodium glutamate on the serum estrogen and progesterone levels in female rat and prevention of this effect with diltiazem. *J. Ayub. Med. Coll. Abbott*. **26**(1), 18–20 (2014). 25358208

[38] Ibekwe H (2019). Effect of methanolic crude extract of Aframomummelegueta (A.m) seeds on selected lactogenic hormones of Albino rats. Int. J. Biochem. Mol. Biol..

[39] Butler, L. G. Effects of condensed tannin on animal nutrition. In *Chemistry and significance of condensed tannins* 391–402, 10.1007/978-1-4684-7511-1_24 (1989).

[40] Aguerre M (2016). Effect of quebracho-chestnut tannin extracts at 2 dietary crude protein levels on performance, rumen fermentation, and nitrogen partitioning in dairy cows. J. Dairy. Sci..

[41] Idoko AS (2015). Growth performance of rats maintained on citrullus colocynthis seed coat-based diet. J. Biotech. Biochem..

[42] Frutos P (2004). A review. Tannins and ruminant nutrition. Span. J. Agric. Res..

[43] Gruendel S (2006). Carob pulp preparation rich in insoluble dietary fiber and polyphenols enhances lipid oxidation and lowers postprandial acylated ghrelin in humans. J. Nutr..

[44] Kato CG (2017). Inhibition of α-amylases by condensed and hydrolysable tannins: focus on kinetics and hypoglycemic actions. Enzym. Res.

[45] Gin H (1999). Effects of red wine, tannic acid, or ethanol on glucose tolerance in non—insulin-dependent diabetic patients and on starch digestibility in vitro. Metabolism.

[46] Anderson RA, Polansky MM (2002). Tea enhances insulin activity. J. Agric. Food Chem..

[47] Sieniawska, E. Activities of tannins—from in vitro studies to clinical trials. *Nat. Prod. Commun*. **10**(11). 10.1177/1934578X1501001118 (2015). 26749816

[48] Goyal RK, Snehal SP (2011). Cardioprotective effects of gallic acid in diabetes-induced myocardial dysfunction in rats. Pharm. Res..

[49] Kim H (2014). Dietary supplementation of chardonnay grape seed flour reduces plasma cholesterol concentration, hepatic steatosis, and abdominal fat content in high-fat diet-induced obese hamsters. J. Agric. Food. Chem..

[50] Roychoudhury S (2017). Potential role of green tea catechins in the management of oxidative stress-associated infertility. Reprod. Bio. Med..

[51] Lipiński K (2017). Polyphenols in monogastric nutrition—a review. Ann. Anim. Sci..

[52] Yang B, Liu P (2014). Composition and biological activities of hydrolysable tannins of fruits of phyllanthusemblica. J. Agric. Food. Chem..

[53] Allam G (2016). Ellagic acid reduces murine schistosomiasis mansoni immunopathology via up-regulation of IL-10 and down-modulation of pro-inflammatory cytokines production. Immunopharmacol. Immunotoxicol..

[54] Marzo F, Tosar A, Santidrian S (1990). Effect of tannic acid on the immune response of growing chickens. J. Anim. Sci..

[55] Zhao MD (2019). Effect of tannins and cellulase on growth performance, nutrients digestibility, blood profiles, intestinal morphology and carcass characteristics in Hu sheep. Asian-Austr. J. Anim. Sci..

[56] Afsana K (2004). Reducing effect of ingesting tannic acid on the absorption of iron, but not of zinc, copper and manganese by rats. Biol. Sci. Biotechnol. Biochem..

